# Campylobacteriosis and Control Strategies against *Campylobacters* in Poultry Farms

**DOI:** 10.4014/jmb.2311.11045

**Published:** 2024-01-15

**Authors:** Mohamad Fadzirul Anwar Zainol, Mansur Bala Safiyanu, Saleha Abd Aziz, Abdul Rahman Omar, Kuo Pin Chuang, Abdul Razak Mariatulqabtiah

**Affiliations:** 1 Department of Cell and Molecular Biology, Faculty of Biotechnology and Biomolecular Sciences, Universiti Putra Malaysia, 43400 UPM Serdang, Selangor, Malaysia; 2 Department of Science Laboratory Technology, School of Science Engineering and Technology, Federal Polytechnic Daura, P.M.B 1049, Daura, Katsina State, Nigeria; 3 Department of Veterinary Pathology and Microbiology, Faculty of Veterinary Medicine, Universiti Putra Malaysia, 43400 UPM Serdang, Selangor, Malaysia; 4 Laboratory of Vaccine and Biomolecules, Institute of Bioscience, Universiti Putra Malaysia, 43400 UPM Serdang, Selangor, Malaysia; 5 International Degree Program in Animal Vaccine Technology, International College, National Pingtung University of Science and Technology, Pingtung 912, Taiwan; 6 Graduate Institute of Animal Vaccine Technology, College of Veterinary Medicine, National Pingtung University of Science and Technology, Pingtung 912, Taiwan; 7 School of Dentistry, Kaohsiung Medical University, Kaohsiung 807, Taiwan; 8 School of Medicine, Kaohsiung Medical University, Kaohsiung 807, Taiwan; 9 Companion Animal Research Centre, National Pingtung University of Science and Technology, Pingtung 912, Taiwan

**Keywords:** Campylobacteriosis, *Campylobacter*, *C. jejuni*, poultry, vaccine

## Abstract

Campylobacteriosis is a significant foodborne illness caused by *Campylobacter* bacteria. It is one of the most common bacterial causes of gastroenteritis worldwide, with poultry being a major reservoir and source of infection in humans. In poultry farms, *Campylobacters* colonize the intestinal tract of chickens and contaminate meat during processing. Vaccines under development against *Campylobacters* in poultry showed partial or no protection against their cecal colonization. Therefore, this review will elaborate on campylobacteriosis and emphasize the control strategies and recent vaccine trials against *Campylobacters* in poultry farms. The epidemiology, diagnosis, and treatment of *Campylobacter* infection, along with specific mention of poultry *Campylobacter* contamination events in Malaysia, will also be discussed.

## Introduction

Campylobacteriosis is a major public health concern and substantial cause of gastroenteritis in humans [1]. The ingestion of contaminated poultry products with *Campylobacter* species serves as a primary mode of transmission, making poultry farms a critical source of the pathogen [2]. Campylobacteriosis is a bacterial infection caused by *Campylobacter* species, primarily *Campylobacter jejuni* and *Campylobacter coli* [3]. It is one of the most common foodborne illnesses worldwide and is associated with the consumption of contaminated poultry products, particularly undercooked or improperly handled chicken and turkey [4].

*Campylobacter* species comprise a group of gram-negative, spiral-shaped, microaerophilic bacteria commonly found in diverse environments [5]. The motility of the bacteria is mediated by the bipolar flagella on each pole and was shown to be a factor for colonization [6]. They are associated with a range of infections in both humans and animals. The primary culprits responsible for human gastroenteritis are *C. jejuni*, causing 80-90% of cases, and *C. coli*, which accounts for 5-10% [7]. Additionally, there exist other *Campylobacter* species, including *C. lari*, *C. upsaliensis*, and *C. fetus*, which can also cause infections in humans, although they are less prevalent than *C. jejuni* and *C. coli* [8]. This diversity of *Campylobacter* species underscores the importance of understanding and managing the risks associated with these bacteria in the context of public health and food safety [9].

## *Campylobacter* Occurrences in Poultry Industries

*Campylobacter* is indeed highly prevalent in poultry worldwide, including various types of poultry such as broilers, layers, turkeys, ducks, and geese. Interestingly, despite its prevalence, *Campylobacter* typically causes little or no clinical disease in poultry [10]. Instead, it is considered a commensal organism that establishes persistent and benign infections in the intestinal tracts of birds [11]. In broilers, for example, colonization levels of *Campylobacter* can reach as high as 1,010 colony-forming units (CFU) per gram of feces [12]. This high level of colonization indicates that *Campylobacter* can thrive and persist in the poultry gut without causing noticeable illness [10]. The shedding of *Campylobacter* by poultry, which has been observed to be highest during the summer and autumn months, can vary depending on various factors, including seasonality. This seasonal variation in shedding may be influenced by environmental and other factors that impact the bird's immune response [13]. It is worth noting that *Campylobacter* is highly prevalent in both commercial poultry production systems and in chickens raised in organic or free-range farms. This indicates that different production systems are equally vulnerable to *Campylobacter* invasion and colonization.

The colonization of poultry by *Campylobacter* primarily occurs in the lower intestinal tract, including the cecum, colon, and cloaca [14]. While the bacterium is most concentrated in these regions, it can also be found in the small intestines and gizzard, and infrequently in the liver, spleen, and gall bladder [10]. *Campylobacter* mainly resides within the mucous layer of the crypts in the intestinal tract and does not directly adhere to epithelial cells. It typically does not cause gross or microscopic lesions, and invasion of the intestinal epithelium is uncommon [15].

*Campylobacter* is considered a normal part of the enteric flora in poultry and does not usually produce clinical disease [16]. Once a broiler chicken becomes infected, it can carry a large number of *Campylobacters* in its intestinal tract and excrete the bacterium in feces for an extended period [17]. The prevalence of *Campylobacter* in poultry increases as the birds grow, reaching its highest levels at the time of slaughter for broiler chickens. It is rarely detected in young birds less than 2 to 3 weeks of age [18]. Once one bird in a flock becomes colonized, the infection rapidly spreads throughout the entire flock within a few days due to high shedding and efficient fecal-to-oral transmission, facilitated by shared access to water and feed [19]. *Campylobacter* colonization of the intestinal tract persists until slaughter, contributing to carcass contamination at the processing plant [20]. High prevalence rates, up to 100% in some cases, can be observed in broilers at slaughter age. Therefore, the practice of feed withdrawal before slaughter can reduce carcass contamination incidences [21].

## Poultry *Campylobacter* Contamination in Malaysia

Isolation of *C. jejuni* from broiler chickens in Malaysia has been performed since 1997 [37]. As the years passed, assessment of *Campylobacter* occurrences in Malaysia has become routine. A more recent study shows that 44% of village chickens and 50% of jungle fowls from a sampled region in Malaysia consisted of *Campylobacters*. These high percentages thus reflect a concern over public health [38]. There was also a report of *Campylobacter* contamination in slaughterhouses which was as high as 61% from 12 modern poultry processing plants in 6 different states in Malaysia. The same study revealed that 80.6% of chicken carcasses prior to washing were contaminated with Campylobacter, which decreased to 62.5% after the inside washing and 38.9% after the post-chilling step [39]. The contamination of *Campylobacter* in Malaysia may occur in fresh produce, such as the local salad vegetable known as Ulam [40], and ready-to-eat sushi [41]. An even more concerning issue is that most of the *Campylobacter* isolated from chicken samples in local Malaysian markets [42] or Malay villages [43] are antimicrobial resistant. One of the possible mechanisms of *Campylobacter* contamination in chickens is via the egg shells. Intact egg shells appear to be permeable to *C. jejuni*, as 4.2% of eggs can be experimentally infected with *C. jejuni* by immersion in a suspension of organisms [44]. This indicates that contact with fecal material could result in egg contamination. However, in eggs experimentally infected in this way, the bacteria are restricted to the inner shell or membranes rather than the egg contents. These results suggest that the natural infection of egg contents, if it occurs, is primarily due to fecal contamination of the external surface and penetration via shell cracks [45]. Moreover, chicks could become orally infected during hatching from such eggshell contamination. Also, a reduction in *Campylobacter* occurrences in poultry farms is expected to decrease the *Campylobacter* transmission to humans.

## *Campylobacters* in Human Host

*Campylobacter* is widely recognized as one of the leading causes of bacterial gastroenteritis in humans worldwide [22]. It holds a prominent position as one of the most prevalent bacterial agents responsible for diarrheal illnesses in many developed nations [23]. Its impact extends to both public health and the economy, as it poses significant challenges in terms of healthcare costs, productivity losses, and food safety concerns. *Campylobacter* species, particularly *C. jejuni* and *C. coli*, are the most common *Campylobacter* species that cause infections in humans [24]. These species are responsible for the majority of Campylobacter-related illnesses worldwide.

*Campylobacter* infections in humans are typically acquired through the consumption of contaminated food, particularly undercooked or raw poultry, unpasteurized milk, and contaminated water [25]. Direct contact with infected animals, particularly their feces, can also lead to transmission [13]. Once ingested, *Campylobacters* can cause gastroenteritis, resulting in symptoms such as diarrhea (often bloody), abdominal pain, fever, and nausea [26]. These symptoms usually start 2 to 5 days after the person ingests *Campylobacter* and last about one week. The infection usually resolves within a week without specific treatment, although in some cases, antibiotics may be prescribed for severe or prolonged infections [27]. It is important to note that while *Campylobacter* infections are generally self-limiting, they can occasionally lead to complications such as Guillain-Barré syndrome, a rare neurological disorder characterized by muscle weakness or paralysis, irritable bowel syndrome, temporary paralysis, and arthritis [28]. In people with weakened immune systems, such as those with a blood disorder, with HIV/AIDS, or receiving chemotherapy, *Campylobacter* can occasionally spread to the bloodstream and cause a life-threatening infection [29].

## Epidemiology of *Campylobacter* Infection

*Campylobacter* species are widespread in nature and can be found in various environments, including animals, water sources, and the environment [30]. It has been estimated that in 2010 *C. jejuni* caused over 95 million foodborne illnesses and 21,000 deaths globally [31]. The main reservoirs for *Campylobacter* are livestock, particularly poultry, cattle, and swine [32]. These animals can carry and shed *Campylobacter* in their feces, leading to contamination of the environment and potential transmission to humans. The incidence of *Campylobacter* infections in humans, termed campylobacteriosis, varies geographically, with higher rates observed in developed countries compared to developing nations [33]. This difference may be due to variations in surveillance systems, reporting practices, and differences in food safety and hygiene practices. Campylobacteriosis can occur throughout the year but peaks during the warmer months, particularly in temperate climates, which may be attributed to increased outdoor activities, consumption of undercooked meats, and potential contamination of food and water sources [33]. The majority of *Campylobacter* infections are sporadic cases, but outbreaks can occur, especially in settings such as nursing homes, hospitals, and childcare facilities [34]. These outbreaks are often associated with the consumption of contaminated food or water, or through person-to-person transmission. Certain population groups are more susceptible to campylobacteriosis, *i.e.*, young children, the elderly, and individuals with weakened immune systems [35]. Travelers to regions with poor sanitation and hygiene practices may also be at an increased risk of acquiring campylobacteriosis [36].

## Diagnosis of *Campylobacter* Infection

*Campylobacter* enteritis can have similar clinical symptoms to other viral or bacterial gastrointestinal illnesses, making it difficult to differentiate based on symptoms alone [46]. Therefore, stool culture is considered as the gold standard for identifying *Campylobacter* species, albeit the isolation using standard culture media can be challenging [47]. One of the reasons is the unique microaerobic conditions with selective antibiotics and a specific gas composition required for bacterial growth [48]. An alternative to this method is through direct examination of the stool sample using contrast microscopy or Gram staining [49], in which *Campylobacters* will appear as curved-or spiral-shaped gram-negative bacteria [5]. However, this method provides a presumptive diagnosis, and confirmation is still required through stool culture.

Polymerase chain reaction (PCR) testing is a molecular diagnostic method that can detect *Campylobacter* from stool samples more frequently than traditional culture-based methods [50]. Studies have shown that PCR testing can identify *Campylobacter* in 20% to 40% more cases compared to culture-based methods [51]. However, it is important to note that PCR tests detect the presence of *Campylobacter* nucleic acid, which may not always indicate the presence of viable organisms or active infection. The clinical significance of a positive PCR result needs to be interpreted in the context of the patient's symptoms, clinical presentation, and other laboratory findings. Another issue with PCR testing is the potential identification of multiple pathogens in a single sample. Co-infections with other bacterial, viral, or parasitic pathogens can occur, making it difficult to determine the specific role of *Campylobacter* in causing the illness. Therefore, in some cases, diagnostic testing may not be necessary for children with acute diarrheal illnesses, as the management may not change based on the specific cause.

## Treatment against *Campylobacter* Infection in Humans

*Campylobacter* infection in humans is usually self-limiting and mild, with symptoms lasting for a few days to 2 weeks [52]. The treatment of *Campylobacter* infection usually involves supportive care by managing symptoms and preventing dehydration. This includes adequate fluid intake to replace fluids lost through diarrhea, rest, and a balanced diet. Oral rehydration solutions or intravenous fluids may be given according to the severity [53].

Antimicrobial treatment can potentially reduce the duration of campylobacteriosis by a day or two, but it is generally not recommended as a routine treatment to alleviate symptoms. Antibiotics may be considered only in severe cases of *Campylobacter* infection or in individuals with certain risk factors, such as young children, the elderly, and those with compromised immune systems [54]. The decision to use antibiotics is typically based on the severity of symptoms, the presence of risk factors, and the potential for complications. It is important to note that there is evidence of increasing resistance to commonly used antibiotics, such as fluoroquinolones and macrolides by *Campylobacter* species [55]. Therefore, the choice of antibiotic may vary depending on local antimicrobial resistance patterns.

## Non-Biological Control Strategies for *Campylobacters* in Poultry Farms

*Campylobacter* control measures at the farm level may include the identification of the most significant risk factors. A typical farm setup such as boot dips, and a reduction in environmental exposure such as via fly screen have been shown to reduce the risk of infection. Increased biosecurity measures including a well-maintained housing system, routine monitoring, thorough cleaning and disinfection, and a limitation of farm access will provide the best benefit in the long term. Non-biosecurity measures such as appropriate treatment of food and water, reduction of slaughter age, and discontinuation of the thinning process are also important to reduce the flock’s susceptibility to infection, or at least delay the disease onset until close to slaughter [56]. Nonetheless, controlling this pathogen from the primary intervention at the housing facilities until the packaging stages has proven to be difficult. Studies showed that various processing stages of chicken carcasses had a substantial impact in reducing *Campylobacter* contamination [57]. For example, freezing the carcasses for 2-3 days or 2-3 weeks will reduce the contamination risk by 50-90% or >90%, respectively. Treatments with tri-sodium phosphate (20%, w/v), citric acid (5 and 10%, w/v) or lactic acid did (5 and 10%, v/v) reduced the total viable *Campylobacter* counts, but not the total Enterobacteriaceae counts in cloacal samples of Campylobacter-infected chickens [58].

## Biological Control Strategies for *Campylobacters* in Poultry Farms

A more sophisticated way to control the *Campylobacter* population is via the use of phage therapy, which uses bacteriophage virus to target the bacteria specifically. *Campylobacter* phages have been isolated from retail poultry, pig, poultry and human feces, sewage, and abattoir effluents [59]. Phages can target a bacterial cell via specific cell surface receptors found on the outer membrane, lipopolysaccharide-binding proteins, and flagella [60]. Campylobacter-targeting phages must be obligatory lytic, *i.e.*, able to infect and reproduce independent of the host DNA genome [61]. They also should be stable in high temperatures, *i.e.*, 42°C, which is the body temperature of live chickens, stable at low pH, *i.e.*, pH 2-4, which is the pH in the chicken gut, and have a well-characterized lytic activity [62]. The lytic activity of a *Campylobacter* phage can be profiled using a panel of reference strains including the wild-type isolates from the same location in which the native host is highly prevalent. Therefore, the *Campylobacter* phages will be confirmed based on their phenotype (via phage typing or microscopic observation), or genotype (via DNA or genomic profiling), and thus represent the actual environmental setting. The phage mechanism of action involves irreversible attachment of the phage on the bacterial cell before DNA ejection which then interrupts the cell’s replication, transcription, and translation processes and eventually kills the cell. During the process, new progeny phages will be assembled in the host cell before being released and infecting other adjacent target cells [61]. Nonetheless, ethical challenges are a major concern in using phages as issues could arise for public health, regulatory challenges, and consent in consumption.

Other non-pathogenic substances that have been tested to control poultry farm *Campylobacters* are probiotics, bacteriocins, and fatty acids. Probiotics from *Lactobacillus* species were shown to reduce *Campylobacter* colonization in mice, invasion into cultured human epithelial cells, release of pro-inflammatory cytokines in culture media [62], and inhibit bacterial growth in vitro and in broiler chickens [63]. Furthermore, a probiotic product made from *Streptococcus faecium* was shown to decrease colonization and shedding of *C. jejuni* in chickens [62]. Probiotics may also synthesize small peptides with antimicrobial activity, termed bacteriocins. A specific bacteriocin, curvaticin DN317, isolated from *Lactobacillus curvatus*, inhibited the growth of *C. jejuni* at a minimum inhibitory concentration (MIC) of 27.3 μg/ml (6.13 nM) [64].

Short-chain and medium-chain fatty acids as poultry feed additives serve as energy sources for gut epithelial cells and possess bactericidal properties. A study by Van Deun and colleagues [65] showed a reduction in *Campylobacter* colonization culture media, but not in broilers, upon supplementation of butyrate in feed. Other medium-chain fatty acids such as capric acid, caprylic acid, caproic acid, and lauric acid have also been used as water additives for *Campylobacter* colonization susceptibility [66]. Similar to butyrate, the in vivo observation of these chemical substances was less promising than the in vitro experiment, thus highlighting the gap between laboratory success and actual conditions in farms.

## Vaccination Against *Campylobacters*

There is no commercially available vaccine against *Campylobacters* for general use so far. However, several vaccine candidates are being explored, and the most common types currently under use are the subunit vaccines (*n* = 21), inactivated vaccines (n =3), and passive immunization (*n* = 1) [67]. These vaccines are aimed at stimulating an immune response against Campylobacter, preventing colonization and infection. One of the main challenges in developing an efficient *Campylobacter* vaccine is the genetic diversity of the bacteria, as there are multiple strains and serotypes [68]. This variability makes it difficult to create a universal vaccine that protects against all *Campylobacter* strains. The identification of common antigens in *Campylobacter* that can be targeted by a vaccine is necessary. Additionally, another challenge faced is the potential for cross-reactivity with other bacteria that are part of the normal gut flora. This could lead to unintended consequences and affect the overall effectiveness of the vaccine [69]. Despite these challenges, progress is being made in *Campylobacter* vaccine development, and clinical trials are underway to assess the safety and efficacy of potential vaccines. Table 1 shows several recent advancements in poultry *Campylobacter* vaccines as the research and development efforts continue.

## Conclusion and Recommendations

An effective *Campylobacter* vaccine that can reduce colonization in caeca of poultry during heterogeneous challenge conditions remains elusive. Therefore, implementing effective strategies to control *Campylobacter* in poultry would directly reduce the incidence of foodborne campylobacteriosis and could also enhance poultry productivity and welfare. Several preventive measures that must be in place, from farm to consumers, are the practice of hand hygiene and safe food and drink handling, avoidance of raw or undercooked poultry meats, and cautionary sanitation during transportation and travelling. Farms and the public must stay informed on food recalls and *Campylobacter* outbreaks in their area in addition to adherence to guidelines from health authorities.

## Figures and Tables

**Table 1:** Recent poultry *Campylobacter* vaccine research and development.

Type of vaccine	Strategy	Findings	Reference
Live attenuated	Live *E. coli* K-12 strain expressing N-glycan from a plasmid and Campylobacter-derived glycosylated outer membrane vesicles was administered orally.	None detectable N-glycan-specific responses and no reduction in *C. jejuni* caecal colonization.	[31]
	Oxidative stress defense knockout mutants ΔahpC, ΔkatA, and ΔsodB derived from *C. jejuni* were administered orally.	ΔahpC and ΔkatA, but not ΔsodB mutants, significantly reduced the level of *C. jejuni* colonization upon high and low doses of challenge after 42 days.	[70]
	Commercial *C. jejuni* strain was deactivated using ciprofloxacin at MIC of 0.012 μg/mL and added to chicken intestinal epithelial cells for invasive assay.	Deactivated *C. jejuni* which could not be recovered was able to adhere and invade chicken intestinal epithelial cells.	Zainol *et al*. (2023)- data unpublished
DNA prime vaccine	Plasmid encoding *C. jejuni* recombinant flagellin A (FlaA) was administered through intramuscular injections.	Although specific systemic and mucosal antibodies were produced, only partial *Campylobacter* loads were reduced. The structure and composition of the caecal microbiota were also affected.	[71]
	Plasmid encoding *C. jejuni* recombinant flagellin A (FlaA) was administered through intramuscular injections.	Despite the production of specific anti-flagellin IgY antibodies and upregulation of the antimicrobial peptide β-defensin, the *Campylobacter* load in the cecum was not reduced.	[72]
Whole-cell autogenous vaccine	*C. jejuni* harboring genes linked to survival outside of the host were isolated and grown in a fermenter before intramuscular injection.	Isolates harboring extra-intestinal survival genes did not reduce *Campylobacter* caecal populations but lowered the populations on the neck skin samples.	[73]
Killed and subunit vaccine	A killed bacterial (bacterin) mix which comprised of 13 *Campylobacter* strains and a subunit vaccine consisting of 6 immunodominant *Campylobacter* antigens were injected intramuscularly.	A prolonged presence of anti- *Campylobacter* antibodies was observed in the serum and intestinal mucus of chicks.	[74]
